# Lost in transition? Professional perspectives on transitional mental health services for young people in Germany: a qualitative study

**DOI:** 10.1186/s12913-018-3462-6

**Published:** 2018-08-22

**Authors:** Sabine Loos, Naina Walia, Thomas Becker, Bernd Puschner

**Affiliations:** Section Process-Outcome Research, Department of Psychiatry II, Ulm University, Bezirkskrankenhaus Günzburg, Ludwig-Heilmeyer-Str. 2, D-89312 Günzburg, Germany

**Keywords:** Transition, Adolescents, Young people, Qualitative research, Mental health services, Professionals, Group discussions

## Abstract

**Background:**

The transition of young patients from child and adolescent to adult mental health services often results in the interruption or termination of care. At this intersection, mental health professionals function as gatekeepers between systems, and their personal views on current clinical practice can contribute to a broader understanding of procedures and help identify reasons for service gaps. This qualitative study investigated the views of mental health professionals on services for young people during the transition from child and adolescent to adult mental health care, as well as on factors which facilitate or hinder continuity of care.

**Methods:**

Four group discussions with 24 mental health professionals with various backgrounds were conducted. Groups were audio-taped, transcribed verbatim and analyzed following the reconstructive approach of R. Bohnsack’s documentary method.

**Results:**

A main theme and six subthemes emerged. Participants’ overall concern was an increasing lack of patient centeredness in care provision. They criticized the limited flexibility and time constraints of their work, which was held to be incompatible with the time-consuming process of engaging young patients in care and coping with their individual needs. A lack of adequate interprofessional exchange and networking was seen as resulting in a diffuse sense of responsibility and a lack of clarity for all involved parties. Participants focused on the adverse impact of neglecting developmental characteristics in care procedures for young patients and revealed personal issues they experienced in their work with young patients (e. g. personal difficulties with diagnosing).

**Conclusions:**

Mental health professionals at this transitional point face a number of complex tasks as well as limitations in terms of time and personal support. An emphasis should be placed on forming and maintaining partnerships within and between systems which could contribute significantly to relieving professionals’ workload. Furthermore, an open style of communication to engage young patients in care is essential. Strengthening communicative skills, improving knowledge about this life stage (especially when working in adult services), and promoting interprofessional encounters can help to develop new procedures in clinical practice. On higher system levels, heightened awareness of the need to reduce fragmentation of care and administrative barriers is needed.

**Electronic supplementary material:**

The online version of this article (10.1186/s12913-018-3462-6) contains supplementary material, which is available to authorized users.

## Background

Transitioning between systems of care runs the risk of interrupting or terminating care. In recent years, young people aged 16–25 with mental health problems and their care needs have attracted increased attention in research and clinical practice [1]. The prevalence rates for mental disorders in this life period suggest a high demand for care [2, 3]. However, compared to all age groups across the life span, young people with mental health problems have the lowest access rates to mental health care [4], and there is a decline in the utilization of mental health care among this age group [5]. Not receiving professional health care when needed has far-ranging negative effects, e.g. regarding educational and professional attainment, social integration, and economic outcomes [6, 7].

A fragmented organization of mental health services for young people increases the risk of discontinuity of care [8, 9]. A smooth institutional transfer for young people with ongoing care needs is often unsuccessful because clinicians fail to refer patients from child and adolescent mental health services (CAMHS) to adult mental health services (AMHS), or because young people refuse to be referred to adult care [10]. Effective programs for transferring to AMHS are rare and face organizational barriers [11]. Differences in care philosophy between CAMHS and AMHS may impede collaboration, and transfer activities may be restricted to personal efforts of individual professionals [12, 13]. In order to implement young people’s own ideas of care, clinicians in the UK recommend better funding and more flexible procedures and policies [14].

Germany has a public, multi-payer health care system (health insurance companies, community organizations or federal states). Mental health care provision is distributed among different sectors and characterized by considerable regional differences. Since 2009, health insurance has been mandatory for the whole population, either in public (non-profit) or private health insurances. Insurances are obliged to provide a broad service package for the treatment of mental disorders, including in- and outpatient care and outpatient psychotherapy restricted to three therapy types (Cognitive-Behavioral Therapy, Psychodynamic Psychotherapy, and Psychoanalysis). The official age boundary for the transition to adult care is 18 years with various exceptions in clinical practice. However, finding the right treatment for young people with mental health problems is challenging. Furthermore, the service transition from child and adolescent to adult care coincides with the vulnerable transition to adulthood with multiple developmental challenges and high demands for supportive alliances [15, 16]. Young people who also struggle with chronic mental health problems are less prepared than their peers to take responsibility due to delayed identity development and increased rumination [17].

Some qualitative studies have investigated the views and experiences of professionals working at this juncture of care provision, predominantly with interviews [13, 18–21]. For example, the TRACK study in the UK explored the views of 34 mental health professionals in semi-structured interviews with a focus on organizational aspects of transition [22]. Professionals described differences between CAMHS and AMHS with respect to service culture and delivery as well as in communication and working practice [13]. CAMHS were described as more family-oriented with a holistic approach whilst AMHS focus more on the individual user, and staff was regarded as lacking confidence in managing young patients. Furthermore, different eligibility criteria and a lack of resources which negatively influenced continuity of care were emphasized [18] In another study from the UK, 39 managers and practitioners were separately interviewed and described mental health services provided for older adolescents (16–19 years) as not user-friendly and unresponsive to young people’s complex service needs; participants also criticized communication deficits at the service planning level and the fact that transfer arrangements were rarely made [21]. Healthcare professionals from primary care (*N* = 37) were interviewed about their screening practice and early interventions in youth with mental health problems in Ireland. They suggested strategies for practice, such as training, raising awareness, closer interprofessional collaboration and a continuous focus on youth engagement [19]. To our knowledge, the only focus group study was carried out in Sweden with 23 professionals from CAMHS and AMHS. It was found that participants focused on two aspects of the transition process: the developmental transition (young adults taking responsibility for themselves and for their care) and the organizational transition (challenges due to different care cultures connected to a need for cooperation as a precondition for secure transition [20]).

In summary, there is a clear need to promote a joint interdisciplinary discourse among mental health professionals in CAMHS and AMHS about strategies to optimize care provision for young people with mental health problems. Research should contribute to a deeper understanding of obstacles to a smooth transition and of good practice from the perspective of health professionals. Group discussions are a suitable method to investigate the views and experiences of participants who share a common social context. However, this method has rarely been applied in this field.

The purpose of this study was to use group discussions to explore the personal experiences of professionals from CAMHS and AMHS with different backgrounds. The research questions were:

1. How do mental health care providers perceive and evaluate mental health services during the transition of young people with mental health problems from CAMHS to AMHS?
2. Which factors facilitate or hinder continuity of care?

## Methods

The study follows a qualitative-explorative and reconstructive approach to gain insight into action-guiding, common or tacit knowledge of groups on the basis of anecdotes or beliefs [23]. The method of group discussions used here is particularly appropriate to explore milieu-specific structures, collective experiences and to develop group opinions [24]. Group discussions, as compared to focus groups, are less structured, have a more non-directive, narrative, and open structure and are guided by the individual group dynamic. The present paper follows the COREQ (Consolidated criteria for reporting qualitative research) checklist [25].

### Research team and reflexivity

The research team consisted of three female researchers (SL, NW and a student assistant, IT, see acknowledgements) who all have training and experience in conducting qualitative research and group discussions. The research team was not involved in patient care and did not know the participants prior to study inclusion. The researchers explained their personal positions in the team and their interest in the research topic to participants (what were the perceived clinical and research gaps, how the research idea was developed, why it was important to the researchers to involve experienced professionals prior to the development of interventions).

### Sampling method and recruitment

We followed a criterion-based sampling method according to purposeful sampling [26]. Groups were arranged according to participants’ area of expertise (either psychiatrists/psychologists or other professions) and current working area (either CAMHS or AMHS).

Study recruitment took place in rural and provincial regions in Germany and in the following settings: at the in- and outpatient services of two Departments of Psychiatry and Psychotherapy at Ulm University in Southern Germany, one for children and adolescents (Ulm) and one for adults (Günzburg); at outpatient outreach clinics for families, children and adolescents in Ulm; and at a community mental health clinic for children and adolescents in central Germany (Herborn). Participants were approached via flyers, personal communication, telephone contact, and by presenting the study at staff conferences or team meetings. Participants gave informed written consent after study information had been provided. The study was approved by Ulm University’s Ethics Committee. Participants were eligible when they had professional contact and experiences with transition-aged youth and young adults with mental health problems. Recruitment took place between September 2015 and July 2016.

### Development of the interview guide

A flexible interview guide with open-ended questions was developed in an iterative process in the research team. Topics (and examples of questions and prompts) were provided for the discussion. A flexible application of the guide allowed the moderator to individually adapt to the specific dynamics of each group discussion. The ideal course of the discussion was intended as follows: (1) warming up, (2) main interview phase including queries by the moderator in exploring themes raised by participants, (3) phase of introducing relevant research topics which had not previously been addressed by group members, (4) confrontation phase, where contradictions, impressions or interpretations are raised by the moderator, and (5) conclusion phase. The interview guide was discussed and revised in a multidisciplinary interpretation group at the research unit of Ulm University’s Department of Psychiatry II (for complete interview guide, see Additional file 1: Table S1).

### Measures

Beside the interview guide, we did not use other standardized materials. Socio-demographic data were obtained using a self-developed form.

### Data collection

Group discussions took place either in a neutral setting at our research division or in the clinical setting. The groups were led by a moderator (NW) and an observer (either IT or SL) who took field notes (e.g. on the seating plan, atmosphere, and non-verbal communication). The moderator’s techniques during the discussion were to listen to the discussion, create an information-eliciting atmosphere and to recognize central topics of shared experiences within the group. If verbal communication stopped among participants, the moderator encouraged further discussions by repeating or reframing a question or by referring to the interview guide.

### Data analysis

Group discussions were audio-taped and transcribed verbatim. Anonymous code names were given to each participant. Participants were invited to inspect the transcript. Data analysis was based on the reconstructive approach of R. Bohnsack’s documentary method [24]. The documentary method concentrates on the reconstruction of experiential knowledge and on factors that implicitly guide our action in practice. The method helps understand explicit and implicit knowledge shared in the group by reconstructing how participants relate to each other and by considering functional aspects in group interactions. Analysis followed a series of steps: (1) transcripts were read independently by team members and the content of each transcript was structured into meaningful paragraphs labelled with open, descriptive headings for themes and sub-themes. (2) In a mutually consensual process, relevant paragraphs were selected for further analysis on the basis of their interactive intensity and thematic relevance. (3) For each selected paragraph, an independent discourse analysis was conducted by two researchers to paraphrase and structure the course of discussion, and individual analyses were compared afterwards. (4) The constant comparison technique was used to compare the meanings of paragraphs within and between groups to extract group consensus. Alternative interpretations and overall impressions and theorizations of the researchers were taken into account. (5) The consensus themes and categories were coded and transferred to an overall coding tree. In its final version, this tree had an interrelated network structure with four levels. It was continuously validated and extended during the process of data analysis. Regular validation sessions were held with an external, multidisciplinary interpretation group to independently discuss preliminary results in the light of various professional perspectives. Subsequent citations were translated into English by SL and approved by the co-authors [27].

## Results

Four group discussions were conducted, lasting between 87 and 98 min. Each. A total of 24 participants took part in the study (*N* = 10 from CAMHS, *N* = 14 from AMHS). On average, professionals were 43.54 (11.93) years old, two thirds were female, and they reported a wide range of working experiences in different professional fields (Table 1).

**Table 1:** Sample description (*N* = 24)

Characteristics	Values
Age, years; M (SD)	43.54 (11.93)
Sex (female, N, %)	14 (58.3%)
Profession	
Psychologist	5 (20.8%)
Psychiatrist	4 (16.7%)
Social worker	4 (16.6%)
Nurse	2 (8.3%)
Medical education nurse	2 (8.3%)
Occupational therapist	2 (8.3%)
Child care worker	2 (8.3%)
Educator	1 (4.2%)
Art therapist	1 (4.2%)
Alternative practitioner	1 (4.2%)
General working experience in mental health care	
< 1 year	1 (4.2%)
1–5 years	3 (12.5%)
6–10 years	12 (50.0%)
11–15 years	1 (4.2%)
> 15 years	6 (25.0%)
Experience working with young patients^a^	
always	7 (29.2%)
frequently	15 (62.5%)
seldom	1 (4.2%)

^**a**^Missing = 1

**Fig. 1:**
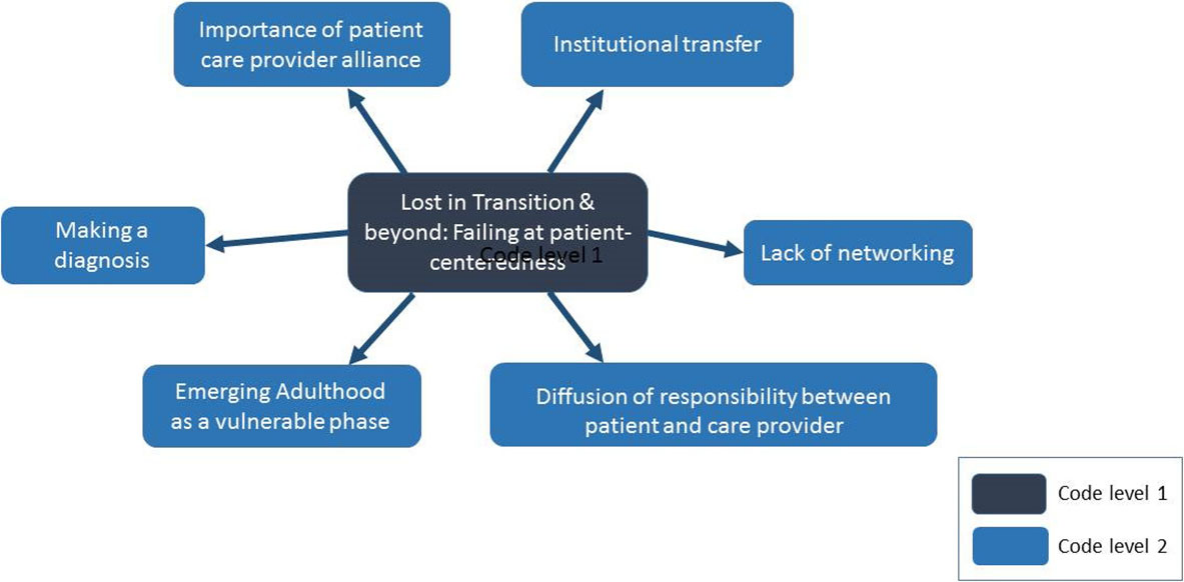
Coding tree as a mind map with two levels

Overall, we extracted 33 codes in a hierarchical structure at four levels with a mixture of descriptive and conceptual codes. Figure 1 shows part of the coding tree as a mind map, a graphical solution including the codes at level 1 and 2 (for the full coding tree, see Additional file 2: Figure S1). The overarching main theme (“Lost in Transition & beyond: failing at patient-centeredness”) and six subthemes were identified at level 2. These themes will be described below.

### Failing at patient centeredness (main theme)

A lack of patient-centered care was a major concern among discussants. Participants mentioned having only limited flexibility of action within rigid system boundaries. These barriers were viewed as impeding the adaptation of care to the individual needs of young people. The current situation in the system was regarded as dominated by economic aspects, with patients considered a “cost factor” instead of individuals with special health care and social needs. This was emphasized as a major impediment to adequately capturing and addressing the frequently complex situation of a young patient.

*At this point, the health care system should engage more. Because it cannot just come from staff. It also has to be funded. And a willingness to see the target population as special has to be developed. And I think there needs to be more of a change in thinking. Health insurance companies themselves should come and say: Hey, what is good now for that patient. And not always cheap, cheap, cheap. It causes immense costs afterwards if people are not continuously treated (male health care professional from CAMHS).*

*How do we create living environments for people who are different? Nowadays, it is more like: How do we place people who are different in already existing living environments, as cost-effectively as possible? (male health care professional from AMHS)*

### Emerging adulthood as a vulnerable phase (subtheme 1)

In general, participants discussed this phase in the life of a young person with increasing demands and stress caused by social pressure to perform. Mental health problems were seen as a logical consequence of these tough demands, and young people with chronic mental health problems were seen as being confronted with duplicate pressures: (a) of finding their way into adulthood and (b) of dealing with their special circumstances.

*Nowadays, social development is like this: Nobody wants to become an adult anymore because being an adult somehow has negative connotations. Where are we now, how do I perceive my role and what are my tasks now; and what should I really deal with? That’s what has been dissolved, and unclear or vanishing structures perfectly correlate with mental health disorders and confusion: Searching and not knowing, reaching for something that is not there anymore (female health care professional from AMHS).*

### Making a diagnosis (subtheme 2)

Care providers from both systems agreed that the process of making a diagnosis for young people with mental health problems is challenging because of the far-ranging consequences of such a diagnosis for the future of a young person. Furthermore, it was reported that making a diagnosis is time-consuming and patients and carers often provide insufficient information.

*It’s very difficult when vague symptoms are present… cognition may be affected or there is depressed mood… when it’s not so distinctive. Then one has to decide, are these simply signs of stress, are these warning signs, or is it just an adolescent crisis… because I think it’s so difficult to distinguish in this ag group, what corresponds to psychotic symptoms and what clinical picture corresponds to a pathological finding* (*male health care professional from AMHS)*.

### The importance of patient-care provider relationship (subtheme 3)

Participants from CAMHS emphasized their stable, long-lasting treatment relationships with young patients as a stabilizing factor. In their experience, adolescents often seem reluctant to begin a new treatment episode in an unknown environment. One reason given for this perception was that transfer falls at a time when people’s lives are characterized by self-doubt, precisely when the opposite is needed. Furthermore, care providers from CAMHS saw potential in functioning as an authentic role model when disclosing personal life changes and handling difficult situations.

*… feedback from some adolescents who say: “Well, there, I have to tell everything from the beginning and I don’t know them at all. Maybe I don’t like them. Maybe there’s no trust”. This is what adolescents tell you from time to time without asking. This is connected with distress and resistance, with everything (male health care professional from CAMHS).*

### Diffusion of responsibility between patient and care provider (subtheme 4)

As a general perception of healthcare at the interface between CAMHS and AMHS, participants noted an increasing tendency among young people and their families to not take responsibility for their care. An overall perception of a lack of willingness to become involved with inner processes and particularly with their own negative feelings was described. Instead, professionals experienced a shift of responsibility directed towards the health care system and its representatives, in contrast to personal capacities and self-efficacy, which are often neglected. A general lack of health literacy despite easy access to information was mentioned.

*How did the treatment contract with us change recently? I increasingly have the feeling that patients or relatives drop the patient concerned off at the clinic like you take a car to a garage. Fix it, please. And if the result doesn’t fit there’s criticism or else they change the professional. But basically it has nothing to do with me… please delete my depression (male health care professional from AMHS).*

### Lack of networking (subtheme 5)

Care provider from CAMHS stated a lack of interest in cooperation and collaborative care planning among AMHS staff during and after the transition process, and a generally low level of interest in young people’s mental health issues. Overall, differences in attitudes between CAMHS and AMHS staff regarding adequate care provision, autonomy and independence was obvious.

*We are part of the service interface but where the other side, that iscolleagues from AMHS are concerned, we actually have no communication with them. Not even on a professional level (female health care professional from CAMHS).*

Closer collaboration with CAMHS was not a subject of discussions among care provider from AMHS. Efforts from CAMHS concerning transfer of patients were only marginally perceived with a negative appraisal. Possible benefits of an active exchange were not addressed.

*As soon as a youngster became 18, they [=CAMHS] rigorously moved the kid to our institution; because of tremendous spatial constraints… here, only the date of birth counts (female health care professional from AMHS).*

### Institutional transfer (subtheme 6)

Participants in all groups agreed that the fixed age boundary of 18 years for transfer from CAMHS to AMHS is not helpful. CAMHS staff pointed out that a well-established and trusting relationship has to be abruptly cut off at a time when no comparable alternative has yet been developed. Furthermore, the transfer of patients was seen as connected with a certain loss of control and feelings of uncertainty about their patients’ futures. Professionals from both CAMHS and AMHS attested to young people’s passive help-seeking strategies.

*… and it’s also difficult for me because I anticipate that they will not be treated as adolescents there but as adults. And as far as I know AMHS, it is much colder and faster, more distanced and medical… (female health care professional from CAMHS).*

## Discussion

This study examined the views of mental health professionals on the transition of young people with mental health problems from child and adolescent to adult mental health services in Germany, with a special focus on factors that hinder or promote continuity of care. To our knowledge, it is the first of its kind within the German mental health system.

A large part of the group discussions was focused on organizational factors and communicative constraints and differences determined by the care system which, in the view of participants, often impeded their clinical work. In particular, financial constraints were mentioned. Thus, service systems in Germany should consider a transition period of parallel care from both CAMHS and AMHS to achieve a “warm and smooth handoff”. This model would imply higher costs in the short-term but might achieve cost savings in the long term. Professionals from CAMHS stated their wish for more exchange and networking regarding young patients’ interests and care needs while, surprisingly, AMHS professionals did not highlight this topic. McLaren and colleagues have referred to a “cultural divide” which was also revealed in qualitative interviews with health professionals in England [13]. These diverging views from representatives of the different care systems point to the importance of harmonizing service interfaces for the benefit of young patients. Information exchange and mutual understanding of practice has also been considered to be a first step towards such harmonization [18]. Furthermore, study participants reported that they experienced a diffusion of responsibility when informal carers (such as family) and other professionals (such as youth welfare workers) became involved in treatment. These findings confirm results from other studies that have identified differences between CAMHS and AMHS with regard to service culture and working practice (i.e. family-oriented vs. individual-oriented care [13, 20]), a lack of understanding and inadequate efforts at cross-collaboration [19] and a lack of standardized transfer protocols or arrangements [21, 28]. Also, young people’s taking responsibility for themselves requires a learning process that should be supported by both CAMHS and AMHS in a constructive atmosphere of trust and confidence [20]. Differing working practices in CAMHS and AMHS might be related to a perceived lack of patient-centeredness due to structural constraints within the health care system.

Professionals, in this study, viewed their work as increasingly dominated by economic and time constraints which left little room for personalized and targeted actions. A bureaucratic, age-based institutional transfer, without clear guidelines or action taken to smoothen impediments was held to contravene current knowledge of youth and emerging adult development and to ignore the importance of relationship at this life stage. This accords with previous studies which found that care fails to address the needs of young people [20, 21, 29] and described a lack of clarity about service responsibilities during the process of transition [18].

The neglect, within mental health care settings, of special developmental characteristics of emerging adulthood which include disorientation, inner conflicts and pressures to live up to the perceived high demands of society was a further dominant theme of group discussions. A forced rupture caused by the care system was considered as contributing to destabilization (of both mental well-being and the care process). This finding is in line with previous studies [19, 20], and it calls for a focus on the social pressure acting on young patients and their mental health.

Finally, due to the open format of the discussions, professionals disclosed personal information and perspectives experienced in their work with young patients. Frequently, in reflecting the process of disagnosing young people’s mental health problems, inner conflicts were reported by professionals. While the participating staff focused on diagnoses as a social construct with a strong impact on patients’ personal development as well as far-reaching consequences for the future, they also reported external pressures on their work from both other societal systems and from their patients’ wish to be considered “normal”. There are various contributing factors such as a lack of openness among patients, time constraints on the diagnostic process, and undisclosed attempts at “self-treatment” among patients. Lack of diagnostic attention and referral in primary care settings may be another factor [19]. Furthermore, participants expressed feelings of loss and helplessness regarding what would happen to their patients at the point of transition, and they also suggested they were likely to personally function as role models in discussing personal crises and the handling of difficult periods in their lives. Thus, the potential of a good dyadic relationship was thought to promote engagement and help-seeking. This should be put to use in providing young patients with information and skills to convey a sense of control, security and personal mastery in the transition process [20, 30].

### Implications for research and practice

Mental health care at the point of transition should be subject to review at different levels. This study adds to an understanding of the personal challenges and needs of professionals: limited flexibility and time in their work resulting in a failure to fulfil the demands of young patients, insufficient interprofessional exchange and collaboration, and the experience of a diffuse responsibility and lack of clarity in the patient-professional-dyad as well as in the system during the transition process. Closer cooperation between CAMHS and AMHS professionals is needed. Barriers to communication and misunderstandings could be overcome through joint, interdisciplinary work in case conferences or quality circles. Advanced training programs could strengthen professionals’ capacity for engaging young patients in care, help qualifying clinicians in these specialist fields and develop new standards for a smooth workflow. Such training should take the developmental and clinical characteristics of the transition from adolescence to adulthood, diagnostic skills, special arrangements for care provision, and communicative and relationship issues with young people into account. Specialized curricula and training should be systematically developed, evaluated and implemented by research projects. These steps could overcome some of the barriers addressed in the discussions. In view of the clinicians’ impression that young patients and their families assume too little responsibility in the process of care and recovery, the importance of active patient (and carer) participation in treatment should proactively be addressed by mental health professionals.

### Strengths and limitations

The main strength of the study is its open, narrative approach, which ensured that the group discussions were dynamic through self-monitoring. Implicit knowledge and orientations could thus be accessed and outlined. Furthermore, participants had a wide range of multi-professional backgrounds and expertise. The primary limitation is that the results should be regarded as country-specific due to the unique characteristics of the German health care system. However, findings of similar themes reported from different care systems underline the generalizability of the problems encountered. The geographical settings for recruitment (rural and small-town areas in southern and central Germany) might have had an impact on the results. Clinicians working in urban regions might have added other viewpoints. We sought to reach theoretical saturation, but due to research constraints, in particular the limited time frame to perform the research project; this may not have been fully reached.

## Conclusions

Mental health professionals perceived health care at the intersection of child and adolescent and adult mental health services as little patient-oriented. Professionals also held the view that system restrictions impeded individualized clinical work. They experienced the diagnostic process as difficult in the age group of emerging adulthood. Diagnosing mental health problems was considered to be stressful with immense societal pressure experienced by young clients. Stable therapeutic relationships can promote the willingness to engage in longer-term treatment while a lack of collaborative exchange between the systems and a rigid age boundary ignoring developmental aspects can impede a smooth transition process. Emphasis should be placed on forming and maintaining partnerships within and between systems along with an open style of communication to engage young patients in care when indicated. Strengthening communicative skills, improving knowledge about this life stage (especially when working in adult services), and promoting interprofessional encounters can help to develop new procedures in clinical practice.

## Additional files

Additional file 1: **Table S1.** Interview guide with examples of questions and prompts for group discussions. (DOCX 27 kb)

Additional file 2: **Figure S1.** Full coding tree of the analysis as a mind-map with four levels. (DOCX 130 kb)

## Abbreviations

AMHS: Adult mental health service
CAMHS: Child and adolescent mental health service
COREQ: Consolidated criteria for reporting qualitative research
