# Supplementary material for: Lost in transition? Professional perspectives on transitional mental health services for young people in Germany: a qualitative study
Source: BMC Health Serv Res. 2018 Aug 22;18:649. doi: 10.1186/s12913-018-3462-6 (PMC6104012; doi:10.1186/s12913-018-3462-6)
Supplement: Supplementary file 1 — Table S1. Interview guide with examples of questions and prompts for group discussions. (DOCX 27 kb) [file 12913_2018_3462_MOESM1_ESM.docx]

| Phase of interview | Principal theme  and method | Subtheme  and method | Examples of questions and prompts |
| --- | --- | --- | --- |
| 1. Warming-up | Mental health care situation of young patients (16-25 years) | Spontaneous thoughts/ ideas relating to context  (inpatient, outpatient or other settings) | - *Please tell us about your practical/clinical work with young patients, what comes to your mind?* - *Which thoughts/images/ situations/experiences come to your mind?* - *How would you describe the current situation concerning transition of young patients?* |
| 1. Main interview phase (including queries) | Personal work experience with young patients with mental health problems  Transition phase | Spontaneous thoughts/ ideas relating to daily routines, course of care procedures  Spontaneous thoughts/ ideas relating to situations, own perceptions |  |
| 1. Phase of introducing relevant research topics which have not yet been addressed by the group | Facilitators and barriers of continuity of care ( | Overall  Subsequent treatment  Care utilization  Change | - *What do young patients need in the transition phase?* - *In which areas do they need support?* - *What promotes or impedes continuity of care during the transition process?* - *How do patients solve problems at this interface?* - *Which factors play a role in the transition process? (structural, setting, personal)?* - *What do you think motivates or hinders young patients to continue treatment? What are their expectations?* - *Imagine you could actively shape the procedures of transitioning, what would you suggest, what is important?* - *Which effect does the transition have on the lives of young patients?* |
| 1. Confrontation phase | Provocative comments, highlighting contradictions from discussion, confrontation with impressions and interpretations of the moderator |  | - *I had the impression that… to what extend am I right?* |
| 1. Conclusion phase | Résumé and conclusion |  | - *Is there anything else important, which has to be considered?* - *Further comments/remarks?* |

**Additional file 1: Table S1**. Interview guide with examples of questions and prompts for group discussions
