# Supplementary figures and images for: Lost in transition? Professional perspectives on transitional mental health services for young people in Germany: a qualitative study
Source: BMC Health Serv Res. 2018 Aug 22;18:649. doi: 10.1186/s12913-018-3462-6 (PMC6104012; doi:10.1186/s12913-018-3462-6)

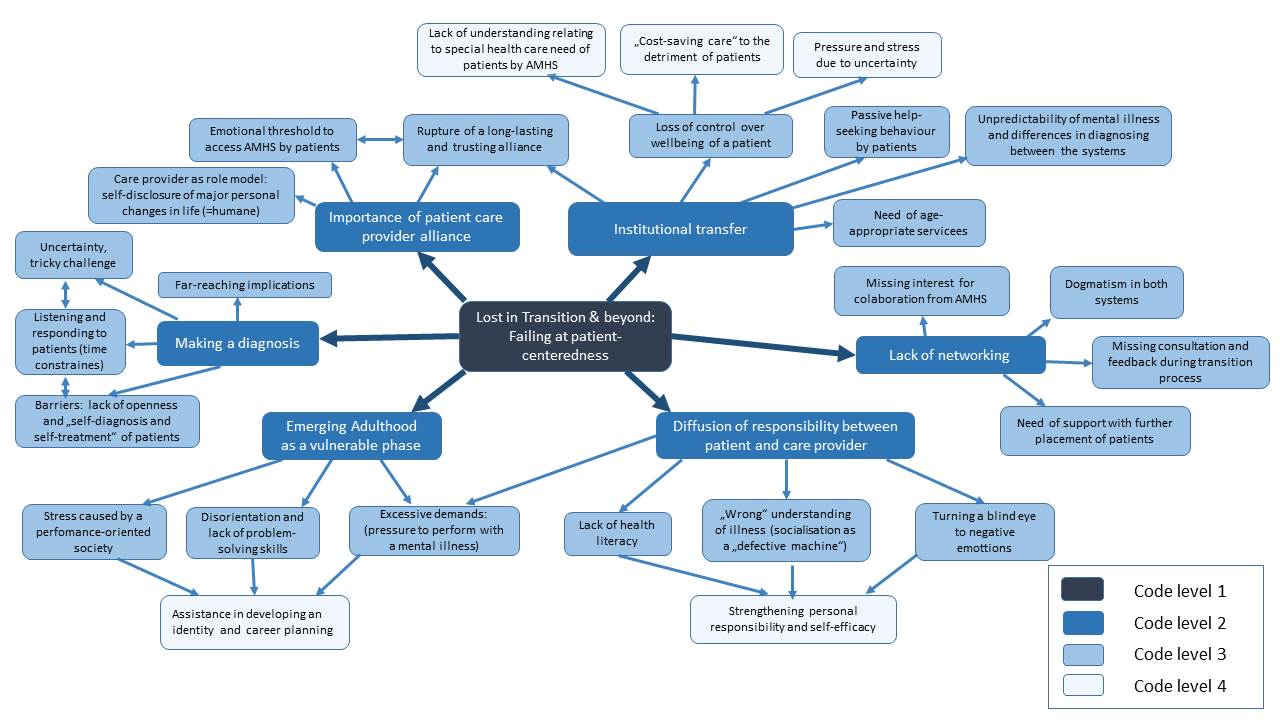


Additional file 2: Figure S1: Full coding tree of the analysis as a mind-map with four levels

Supplement: Supplementary file 2 — Figure S1. Full coding tree of the analysis as a mind-map with four levels. (DOCX 130 kb) [file 12913_2018_3462_MOESM2_ESM.docx]
